# Actinomycin V Inhibits Migration and Invasion via Suppressing Snail/Slug-Mediated Epithelial-Mesenchymal Transition Progression in Human Breast Cancer MDA-MB-231 Cells In Vitro

**DOI:** 10.3390/md17050305

**Published:** 2019-05-24

**Authors:** Shiqi Lin, Caiyun Zhang, Fangyuan Liu, Jiahui Ma, Fujuan Jia, Zhuo Han, Weidong Xie, Xia Li

**Affiliations:** 1 School of Ocean, Shandong University, Weihai 264209, China; lsqsd@outlook.com (S.L.); caiyun617@outlook.com (C.Z.); fangyuan617@outlook.com (F.L.); sdumjh@hotmail.com (J.M.); jfj1996@outlook.com (F.J.); hanzhuo1013@gmail.com (Z.H.); wdxie@sdu.edu.cn (W.X.); 2 School of Pharmaceutical Sciences, Shandong University, Jinan 250012, China; 3 The Key Laboratory of Chemistry for Natural Product of Guizhou Province, Chinese Academy of Science, Guiyang 550002, China

**Keywords:** breast cancer, actinomycin, EMT, migration, invasion

## Abstract

Actinomycin V, an analog of actinomycin D produced by the marine-derived actinomycete *Streptomyces* sp., possessing a 4-ketoproline instead of a 4-proline in actinomycin D. In this study, the involvement of snail/slug-mediated epithelial-mesenchymal transition (EMT) in the anti-migration and -invasion actions of actinomycin V was investigated in human breast cancer MDA-MB-231 cells in vitro. Cell proliferation effect was evaluated by 3-(4,5-Dimethylthiazol)-2,5-diphenyltetrazolium bromide (MTT) assay. Wound-healing and Transwell assay were performed to investigate the anti-migration and -invasion effects of actinomycin V. Western blotting was used to detect the expression levels of E-cadherin, N-cadherin, vimentin, snail, slug, zinc finger E-box binding homeobox 1 (ZEB1), and twist proteins and the mRNA levels were detected by rt-PCR. Actinomycin V showed stronger cytotoxic activity than that of actinomycin D. Actinomycin V up-regulated both of the protein and mRNA expression levels of E-cadherin and down-regulated that of N-cadherin and vimentin in the same cells. In this connection, actinomycin V decreased the snail and slug protein expression, and consequently inhibited cells EMT procession. Our results suggest that actinomycin V inhibits EMT-mediated migration and invasion via decreasing snail and slug expression, which exhibits therapeutic potential for the treatment of breast cancer and further toxicity investigation in vivo is needed.

## 1. Introduction

Breast cancer has the highest diagnosis and death rates among women followed by lung and colorectal cancer and has generally increased in recent years [1]. Since many therapies including surgery, radiotherapy, chemotherapy, and human epidermal growth factor receptor 2 (HER2) targeted therapy have been used in clinical trials, the overall survival rate of patients still remained poor [2]. Metastasis is the majority reason for breast cancer death. Epithelial-mesenchymal transition (EMT) is a key process in cancer invasion and metastasis [3]. EMT is a dynamic and complex multistep process that cells attach to and degrade the surrounding extracellular matrix (ECM) to escape from the primary tumor site, invade the stromal tissues, and establish the distant secondary tumor [4]. Therefore, attention has been focused on blocking the migration and invasion processes of breast cancer cells to inhibit metastasis [5,6,7].

Many effective anti-cancer compounds that were originally isolated from marine invertebrates, such as discodermolide, halichondrin B, bryostatin1, and phorboxazole A, were actually produced by marine microorganisms [8,9,10]. Marine microorganisms were considered to be the promising sources for discovering new effective anti-cancer drugs and many efforts have been made in recent years. Actinomycins, produced by the marine-derived actinomycete *Streptomyces* sp., is a class of cyclic chromopeptide consisting of two cyclic pentapeptide lactones attached to a central phenoxazinone chromophore via amide bonds [11,12,13]. Typically, actinomycin D has been well used in treatment of a variety of cancers such as Wilms tumor, rhabdomycosarcoma, and Ewing’s sarcoma through binding and intercalating double-stranded DNA and inhibiting RNA synthesis [14]. A report has shown that actinomycin D inhibited the migration of MDA-MB-231 cells but the underlying mechanism was not mention [15]. Moreover, the toxicity development, particularly in hepatotoxicity of actinomycin D, is associated with use in an anti-tumor process, which severely limits its effectiveness in clinic. Actinomycin V possesses 4-ketoproline as a substitute for 4-proline of actinomycin D. Studies have reported that actinomycin V’s inhibitory effects on the F5-5 friend leukemia cells, MCF-7, A549, and K562 cells were superior to actinomycin D and the effect on human breast cancer cells MCF-7 was the most obvious [16,17]. However, the anti- migration and invasion actions of actinomycin V and the underlying mechanism remains unclear. In this present study, we examined that actinomycin V treatment inhibited the proliferation of human breast cancer cells and suppressed the EMT process by down-regulating the snail and slug protein expression. These results may provide a new theoretical basis for the treatment of breast cancer with actinomycin V and the useful information for developing novel marine-derived anti-cancer drugs.

## 2. Results

### 2.1. Actinomycin V Inhibits the Proliferation of Human Breast Cancer Cells

**Figure 1:**
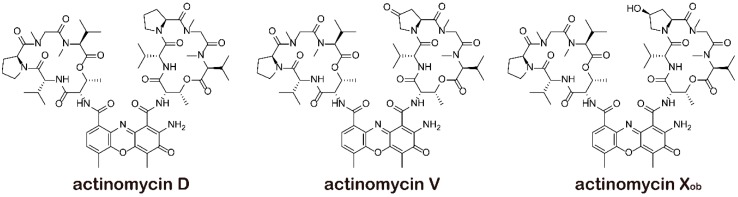
Chemical structure of actinomycin D, actinomycin V, and actinomycin X_ob_.

**Figure 2:**
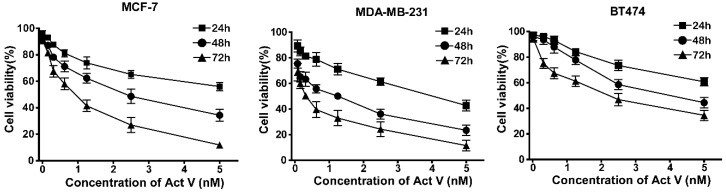
Actinomycin V does- and time- dependently inhibited the proliferation of various cell lines. Cells were treated with various concentrations of actinomycin V (0 to 5 nmol/L) for 24 h to 72 h. Cell viability was denoted as a percentage of the untreated control (actinomycin V 0 nmol/L) at the concurrent time point. Results were obtained from three independent experiments.

To compare the effects of three actinomycins (actinomycin D, actinomycin V, and actinomycin X_ob_ as shown in Figure 1) on breast cancer cells, we initially performed 3-(4,5-Dimethylthiazol)-2,5-diphenyltetrazolium bromide (MTT) analysis in several subtypes of human breast cancer (MDA-MB-231, BT474, and MCF-7) and noncancer (HMLE and MCF-10A) cell lines. We found that actinomycin V had the excellent activity on breast cancer cells among three actinomycins. Actinomycin V displayed the strongest sensitivity on MDA-MB-231 cells and with lower cytotoxicity in noncancer breast cells as shown in the Table 1. The IC_50_ of actinomycin V for 48 h treatment to MDA-MB-231, MCF-7, BT474, HMLE, and MCF-10A were 0.83 ± 0.32 nmol/L, 1.92 ± 0.25 nmol/L, 4.16 ± 0.25 nmol/L, 3.49 ± 0.31 nmol/L, and 4.07 ± 0.26 nmol/L, respectively, and actinomycin D treatment for 48 h to those cell lines were 15.15 ± 0.52 nmol/L, 8.23 ± 0.50 nmol/L, 37.00 ± 3.15 nmol/L, 30.22 ± 0.50 nmol/L, and 34.01 ± 0.25 nmol/L, respectively. Further experiments showed that actinomycin V dose- and time-dependently inhibited the proliferation of human breast cancer cells (Figure 2). 

### 2.2. Actinomycin V’s Effects on Morphological Changes in Breast Cancer Cells

**Figure 3:**
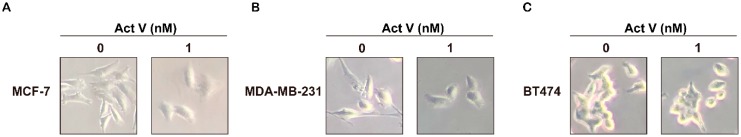
Changes in morphology of MCF-7, MDA-MB-231, and BT474 cells upon actinomycin V treatment. (**A**–**C**) Cells were treated with 1 nmol/L actinomycin V for 24 h and phase contrast images of the cultured cells were taken under 200× objective lenses.

We observed the effects of actinomycin V on morphological changes in breast cancer cells (MCF-7, MDA-MB-231, and BT474) (Figure 3). After 1 nmol/L actinomycin V 24 h treatment cells performed in different levels of morphological changes. Especially the metastatic MDA-MB-231 cells, it displayed in a scattered distribution in the culture and a spindle or star-like structure before actinomycin V treated but after 24 h treatment cells changed to a round cell shape and tend to an aggregation distribution, indicating cells appeared to have improved intercellular adhesion and reduced cells migration and invasion.

### 2.3. Actinomycin V Inhibits the Migration and Invasion of Human Breast Cancer Cells

**Figure 4:**
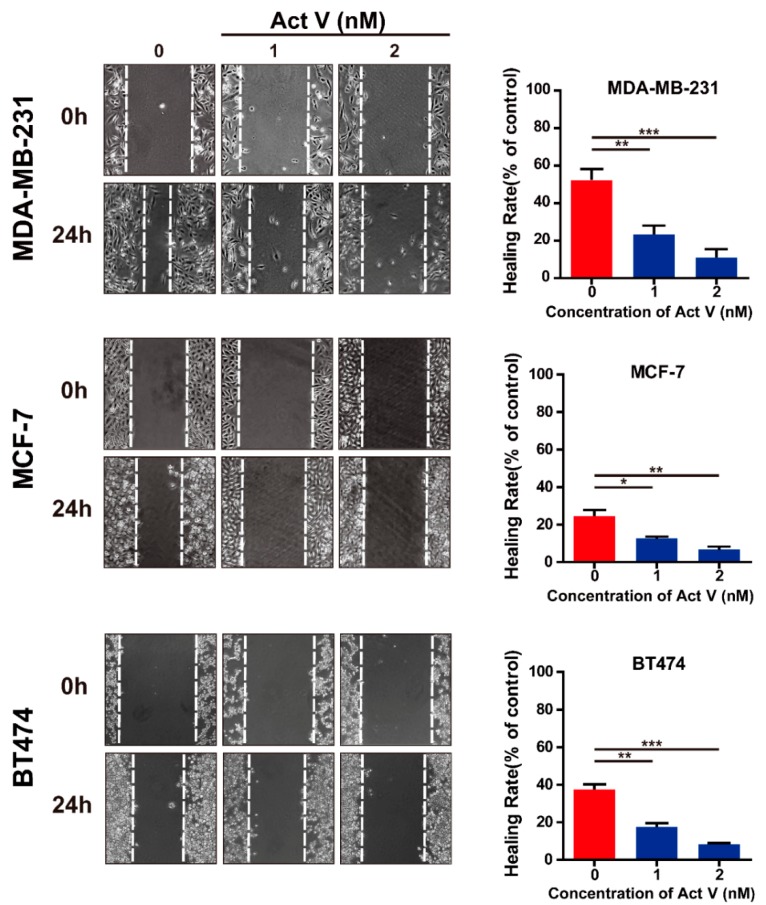
Effect of actinomycin V on the motility of MDA-MB-231, MCF-7, and BT474 cells. The wound-healing rate of each cell line was analyzed (magnification: ×200). Results were obtained from three independent experiments. * *p* < 0.05, ** *p* < 0.01, *** *p* < 0.001.

**Figure 5:**
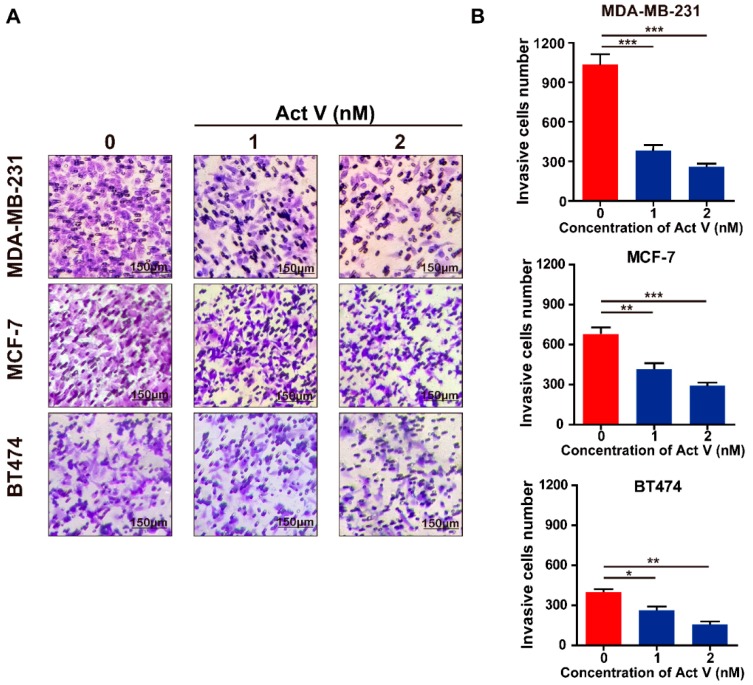
Effects of actinomycin V on the invasion of MDA-MB-231, MCF-7, and BT474 cells. (**A**,**B**) Cells were treated with 0–2 nmol/L actinomycin V for 24 h, and the invasion in these two cell lines was analyzed (magnification: ×200). Results were obtained from three independent experiments. * *p* < 0.05, ** *p* < 0.01, *** *p* < 0.001.

Considering that migration and invasion are the major drivers of breast cancer metastasis, wound-healing assay and Transwell assay were used to further confirm the actinomycin V effects on anti-migration and anti-invasion in human breast cancer cells. In the wound-healing assay (Figure 4), the untreated MDA-MB-231 cells wound-healing rate was 52% in 24 h. However, actinomycin V treatment apparently retarded the wound healing, the healing rates were only about 23% and 10%, respectively, in the presence of 1–2 nmol/L actinomycin V. In the Transwell assay (Figure 5), actinomycin V dose-dependent treatment significantly suppressed the invasion of MDA-MB-231 cells. Similarly, actinomycin V treatment inhibited the migration and invasion of MCF-7 and BT474 cells but the inhibitory effects were not as significant when compared with its anti-migration and anti-invasion effects on the metastatic MDA-MB-231 cells. These results indicated that actinomycin V inhibited the migration and invasion of human breast cancer cells.

### 2.4. Effects of Actinomycin V on the Expression of EMT-Associated Proteins and mRNA in MDA-MB-231 Cells

**Figure 6:**
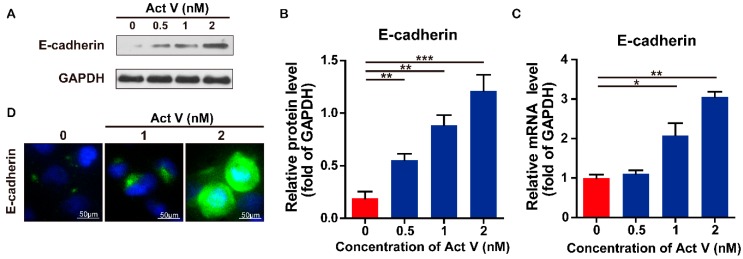
Effect of actinomycin V on the expression of E-cadherin. (**A**,**B**) MDA-MB-231 cells were treated with 0–2 nmol/L actinomycin V for 24 h then the protein expression of E-cadherin was measured by Western blot. (**C**) Relative expression of E-cadherin mRNA in the MDA-MB-231 cells was analyzed by real-time PCR. RNA levels are represented as fold increase relative to the level of the control (normalized to glyceraldehyde-3-phosphate dehydrogenase (GAPDH) mRNA level). Results were obtained from three independent experiments. * *p* < 0.05, ** *p* < 0.01, *** *p* < 0.001. (**D**) cells were treated with 0–2 nmol/L actinomycin V for 6 h and analyzed by E-cadherin fluorescent signals. Cells were stained with anti- E-cadherin (green) and 4′,6-diamidino-2-phenylindole (DAPI, blue). Magnification ×200. E-cadherin levels were increased in the cells, consistent with the Western blot results.

**Figure 7:**
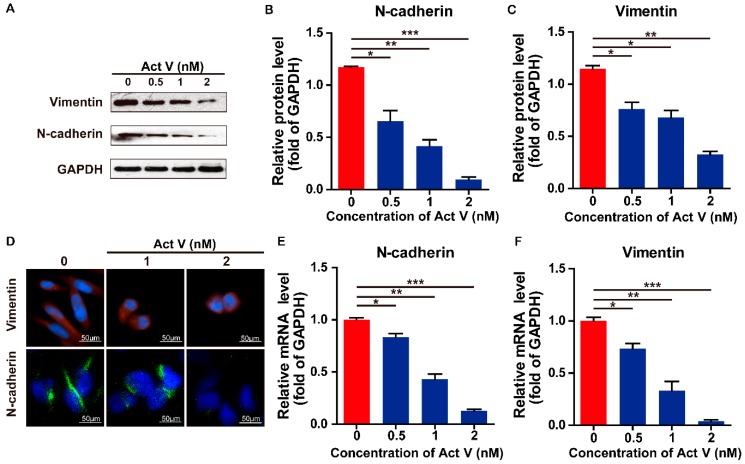
Effects of actinomycin V on the N-cadherin and vimentin expression. (**A**–**C**) MDA-MB-231 cells were treated with 0–2 nmol/L actinomycin V for 24 h then the protein expression of vimentin and N-cadherin was measured by Western blot. (**D**) cells were treated with 0–2 nmol/L actinomycin V for 6 h and analyzed by vimentin and N-cadherin fluorescent signals. Cells were stained with anti-vimentin (red), N-cadherin (green), and DAPI (blue). Magnification: ×200. Vimentin and N-cadherin levels were decreased in the cells, consistent with the Western blot results. Results were obtained from three independent experiments. * *p* < 0.05, ** *p* < 0.01, *** *p* < 0.001. (**E**,**F**) Relative expression of vimentin and N-cadherin mRNA in the MDA-MB-231 cells were analyzed by real-time PCR. RNA levels are represented as fold increase relative to the level of the control (normalized to GAPDH mRNA level).

To investigate the actinomycin V effects on Epithelial-mesenchymal transition inhibition in human breast cancer cells, we preformed the Western blot analysis to evaluate the expression levels of the epithelial marker E-cadherin, and mesenchymal markers N-cadherin and Vimentin in MDA-MB-231 cells (Figure 6 and Figure 7). We found that actinomycin V treatment increased the expression levels of E-cadherin while decreased that of N-cadherin and Vimentin. Immunofluorescence staining also showed that compared with untreated cells, actinomycin V treatment significantly decreased intracellular N-cadherin and vimentin content while increased the E-cadherin content in cancer cells.

Real-time PCR (rt-PCR) showed that the mRNA level of E-cadherin was upregulated while the N-cadherin and Vimentin were downregulated significantly in response to actinomycin V treatment. These results confirmed that actinomycin V inhibited the epithelial-mesenchymal transition process in MDA-MB-231 cells.

### 2.5. Actinomycin V Suppresses the EMT Process by Reducing the Expression of SNAIL and SLUG

**Figure 8:**
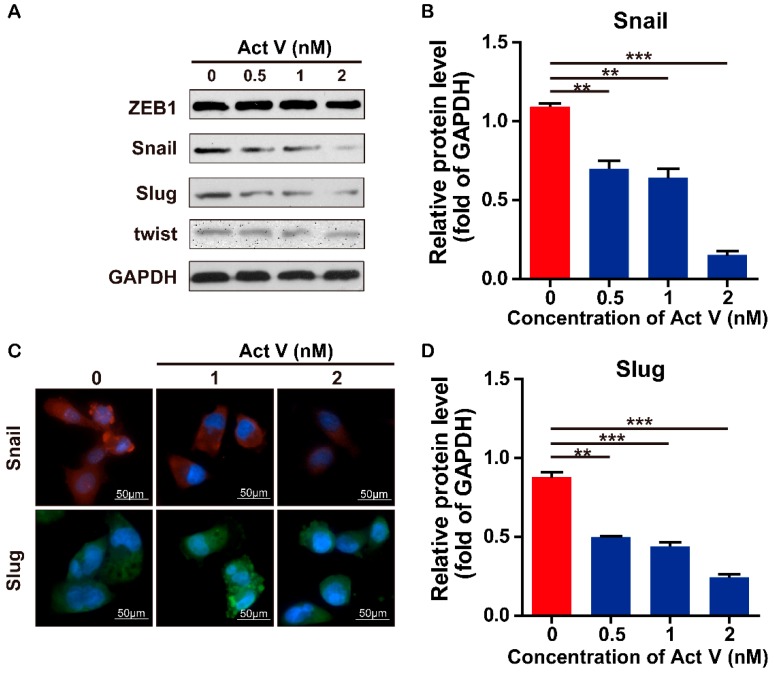
Effects of actinomycin V on the expression levels of snail and slug. (**A**,**B**,**D**) MDA-MB-231 cells were treated with 0 to 2 nmol/L actinomycin V for 24 h then the protein expression of snail and slug was measured by Western blot. Results were obtained from three independent experiments. * *p* < 0.05, ** *p* < 0.01, *** *p* < 0.001. (**C**) cells were treated with 0 to 2 nmol/L actinomycin V for 6 h and analyzed by snail and slug fluorescent signals. Cells were stained with anti-snail (red), slug (green) and DAPI (blue). Magnification ×200. Snail and slug levels were decreased in the cells, consistent with the Western blot results.

EMT is a complex process which required a complicated network of transcription factors such as zinc finger and basic helix loop helix to participate in. Snail family transcriptional repressor 1 (also known as SNAIL) and Snail family transcriptional repressor 2 (SLUG) are proved to be the E-cadherin repressors and act as inducers of the invasion process when they are overexpressed in epithelial cell lines [18,19]. As shown in Figure 8, we found that actinomycin V treatment down-regulated the expression levels of snail and slug protein while the expression levels of zinc finger E-box binding homeobox 1 (ZEB1) and twist remained unchanged in MDA-MB-231 cells. Immunofluorescence staining also showed the same reduction effects after 1–2 nmol/L actinomycin V treatment. Therefore, we suggest the actinomycin V inhibitory effect on EMT process may through down-regulate the expression levels of the snail and slug protein.

## 3. Discussion

The majority reason of the high incidence and death rates of breast cancer among women is metastasis, which is initiated by the processes of adhesion, invasion and migration [20]. First, adhesion, it plays a vital role in maintaining cellular structure and the disruption of cellular adhesion may led to cell invasion and migration [6,7]. Cellular adhesion is mediated by a variety of membrane molecules, for example, cadherins, the Ca^2+^-dependent transmembrane proteins, is one of the major members of adhesion related membrane molecules [21]. More specially, reports have been confirmed that E-cadherin was required to initiate cell-cell adhesion and the procession of epithelial-mesenchymal transition (EMT) is closely related to its deletion [22,23]. In this paper, we found that the actinomycin V was effective in suppressing migration and invasion of breast cancer cells as revealed by morphological changes, wound-healing and Transwell assays. Moreover, actinomycin V treatment upregulated the protein and mRNA levels of E-cadherin, indicating that actinomycin V enhanced cell-cell adhesion in MDA-MB-231 cells. Next, we measured the N-cadherin and Vimentin, the other two important maker molecules of EMT, by Western blotting and immunofluorescent staining. E-cadherin serves as an invasion suppressor, N-cadherin, which is frequently upregulated in cancer cells, functions as an invasion promoter. The expression of N-cadherin induces epithelial cells transform the morphological into a fibroblastic phenotype and the biological function also changes to make epithelial cells more motile and invasive in the EMT process [24]. Vimentin is one of the major members of intermediate filament family and frequently overexpresses in a variety of cancer cells such as prostate cancer, breast cancer and lung cancer. The overexpression of vimentin in cancer cells promotes cellular proliferation, invasion and migration [25]. Our results showed that actinomycin V treatment significantly down-regulated both of the protein and mRNA expression levels of N-cadherin and vimentin, which may inhibit EMT in cells by reducing cells invasion and migration. These findings suggest that actinomycin V inhibits MDA-MB-231 cells migration and invasion via suppressing the EMT process.

A complicated network of transcription factors (TFs) are involved in EMT process. Factors from Snail, ZEB and helix-loop-helix families are acted as the inducers of epithelial-mesenchymal transition (EMT) and potent repressors of E-cadherin expression [26,27]. The activation of these factors initiates the EMT process. Then we examined the effects of actinomycin V on these family members including ZEB1, twist, slug, and snail. We found that the actinomycin V treatment down-regulated the expression levels of snail and slug protein while the expression levels of ZEB1 and twist remained unchanged. Immunofluorescence staining also showed us that the expression of snail and slug were reduced in MDA-MB-231 cells after actinomycin V treatment. It has been reported that snail and slug active during EMT and down-regulate the expression of adhesion genes like E-cadherin. Snail and slug also involved in the expression of the invasion promoter such as vimentin, N-cadherin, and fibronectin [27,28,29,30]. Therefore, the reduction of snail and slug may lead to the up-regulation of E-cadherin expression while the down-regulation of vimentin and N-cadherin and suppress the EMT process, which is consistent with the results showed in our studies. As a result, we conclude that the actinomycin V suppresses EMT process via down-regulating the snail family members snail and slug and subsequently inhibits the proliferation, migration, and invasion of breast cancer cells. On the other hand, it is noticed that the selectivity index of actinomycin V between cancer (such as MDA-MB-231) and noncancer cell lines (HMLE/MCF-10A) still remains low (around 4–5) as shown in Table 1. Toxicity investigation should be especially warranted in vitro/vivo for actinomycin V drug development.

## 4. Materials and Methods

### 4.1. Chemicals and Reagents

Actinomycin V (>98%), actinomycin X_ob_ (>98%) and actinomycin D (>98%) were provided by Dr. Xie (Shandong University, Weihai, China) isolated from *Streptomyces* sp. N1510.2 and identified by ESI-MS, ^1^H and ^13^C NMR data [31]. Compounds were dissolved in dimethylsulfoxide (DMSO) as 10 μmol/L stock solution and diluted according to experimental requirement. MTT (3-(4,5-Dimethylthiazol)-2,5-diphenyltetrazolium bromide) was purchased from Sigma-Aldrich Corp. (St. Louis, MO, USA). Antibodies against E-cadherin, N-cadherin, Vimentin, snail and ZEB1 were purchased from Cell Signaling Technology (CST, Inc., Beverly, MA, USA). Slug, twist, GAPDH antibodies and goat/donkey anti- mouse/rabbit IgG-Alexa Fluor 488/647 secondary antibodies were purchased from Abcam, Inc. (Cambridge, MA, USA). The primers used in this study were bought from Sangon Biotech Co Ltd. (Shanghai, China). All chemicals used in this study were commercial reagent grade products. 

### 4.2. Cell Lines and Cell Culture

Human breast cancer cell lines MCF-7, MDA-MB-231, BT474 and normal human breast epithelial cell lines HMLE and MCF-10A were purchased from the Shanghai Institute for Biological Sciences (SIBS), Chinese Academy of Sciences (Shanghai, China). MCF-7, MDA-MB-231 and BT474 were cultured in RPMI 1640 Medium (Hyclone Laboratories, Inc., Logan, UT, USA) and HMLE cells were cultured in DMEM/F-12 1:1 (Hyclone Laboratories, Inc.) containing 10% fetal bovine serum supplemented with 100 units/mL of penicillin and 100 μg/mL of streptomycin. MCF-10A cells were cultured in DMEM/F-12 1:1 containing insulin, hydrocortisone, EGF and 10% horse serum supplemented with 100 units/mL of penicillin and 100 μg/mL of streptomycin. All cell lines were cultured in a humidified incubator at 37 °C with 5% CO_2_.

### 4.3. MTT Assay

Cell proliferation inhibitory effect of actinomycin V was evaluated by MTT assay. Cells were seed in 96-well plates at a density of 5 × 10^3^ cells per well. After 24 h incubating, cells were treated with actinomycin V, actinomycin X_ob_ and actinomycin D at various concentrations while adriamycin was used as the comparison. After 24–72 h of continuous culture, 15 μL MTT (5 g/L) was added to each well and incubated for another 4 h at 37 °C. The medium with MTT was removed and 150 μL per well of DMSO was added to dissolve the formazan. The absorbance of each well was measured by a microplate reader at 570 nm. IC_50_ values (concentration resulting in 50% inhibition of cell growth) for each cell line were calculated by plotting the untreated cells, which were considered to be 100% cell survival. Each text was performed in triplicate independently.

### 4.4. Wound-Healing Assay

Cells in logarithmic growth phase were seeded into 6-well plates at a density of 3 × 10^4^ cells per well. When the cells had adhered to 90%, they were scratched by micropipette tips vertically in the middle of each well. Each plate was washed with 3 mL PBS three times to remove the suspension cells. The cells in plate were starved for 12 h to eliminate the interference of proliferation in advance and then treated with 0 to 2 nmol/L actinomycin V for 24 h. The scratch width changes of each group were observed under a microscope, and the wound healing rate (%) was calculated as (1 − (scratch width of the actinomycin V group/scratch width of the control group)) × 100% to evaluate the migration ability of cells. Each experiment was performed in triplicate independently.

### 4.5. Transwell Assay

We used transwell assays to measure the invasive capacity of cells. We took 50 μL Matrigel diluted with 1:8 of serum-free culture medium and added onto the upper chamber membrane of the insert. After solidifying into gel, cells were suspended in 200 μL serum-free culture medium at a destiny of 1 × 10^5^ cells/mL and then added into the upper chamber of the insert, while the lower chamber were filled with 600 μL culture medium containing 20% FBS. Cells were treated with varying concentration of actinomycin V ranging from 0 to 2 nmol/L and incubated with 5% CO_2_ at 37 °C for 24 h. The medium in chamber was removed and the cells in the upper chamber were wiped by cotton swabs. Cells in lower chamber were fixed in formaldehyde for 20 min, then stained with 0.5% crystal violet for 15 min. After three times washing, five fields were taken randomly under a Nikon TE2000 microscope (Nikon Instruments Inc., Tokyo, Japan) and the number of transmembrane cells were counted. Each experiment was performed in triplicate independently.

### 4.6. Immunofluorescence Staining

Cells were seeded onto 12-mm round, glass over slips in 24-well plates and treated with 0 to 2 nmol/L actinomycin V for 6 h. Cells were washed and fixed with 4% Paraformaldehyde for 15 min, washed three times with cold PBS, and permeabilized in 0.1% Triton X-100 for 20 min at room temperature. To prevent non-specific antibody binding, the cells were washed and incubated with 3% goat serum for 30 min. Then cells were incubated with the E-cadherin, N-cadherin, vimentin, snail and slug antibody at 4 °C overnight, washed and incubated with donkey anti-rabbit IgG-Alexa Fluor 647 antibody or goat anti-mouse IgG-Alexa Fluor 488 antibody for 1 h. After washing three times, cells were counterstained with 4 μg/mL 4′,6-diamidino-2-phenylindole (DAPI) for 10 min at room temperature. After washing three times with PBS-TX, cover slips containing the cells were then mounted using mounting medium (PBS:glycerol = 1:1 (*v*/*v*)). Fluorescence images were captured by using a fluorescence microscope.

### 4.7. Western Blot Analysis

Cells were treated with indicated concentrations of actinomycin V for 24 h, then the protein from whole cell lysates was analyzed by Western blot assay. Samples were denatured by boiling with 1 × Laemmli buffer, subjected to 10% SDS-polyacrylamide gel electrophoresis and transferred to nitrocellulose membranes. The membranes were washed with distilled water and then blocked with 5% non-fat milk in TBS-T buffer (10 mmol/L Tris-HCl, 150 mmol/L NaCl and 0.05% Tween-20 (*v*/*v*), pH 7.8) for at least 1 h at room temperature. After a short wash in TBS-T buffer, the membranes were incubated in a solution containing monoclonal antibodies specific for GAPDH, Vimentin, E-cadherin, N-cadherin, snail, slug, twist and ZEB1 for 2 h at room temperature or overnight at 4 °C. Then, the membranes were washed with TBS-T buffer for 6 min three times and once in TBS buffer. Membranes were incubated in secondary HRP-conjugated goat anti-mouse IgG or donkey anti-rabbit IgG for 1 h at room temperature. Membranes were washed with TBS-T buffer three times and once in TBS buffer. Proteins on the membranes were visualized using the enhanced chemiluminescence detection system (ECL^®^, Amersham Biosciences). Proteins expression density values were quantified by gray analysis using the Image J 2.0 software (National Institutes of Health, Bethesda, MD, USA).

### 4.8. RNA Extraction and Relative Quantification by Real Time PCR

Cells were seeded in 6-well plates and treated with 0 to 2 nmol/L actinomycin V for 24 h. Total RNA was extracted using RNeasy kit under the manufacturer’s instructions and the purity of RNA was detected by OD260/280 of RNA sample (>1.8). Then we converted the RNA into cDNA using the ReverTra Aoe^®^ qPCR RT Kit (QIAGEN, Hilden, Germany). Primers were designed by primer premier 5 for the E-cadherin gene (forward primer: 5′-CGAGAGCTACACGTTCACGG-3′ and reverse primer: 5′-GGGTGTCGAGGGAAAAATAGG-3′), N-cadherin gene (forward primer: 5′-CCTTTCAAACACAGCCACGG-3′ and reverse primer: 5’-TGTTTGGGTCGGTCTGGATG-3’), and vimentin gene (forward primer: 5′-GACGCCATCAACACCGAGTT-3′ and reverse primer: 5′-CTTTGTCGTTGGTTAGCTGGT-3′). GAPDH gene (forward primer: 5′-CATCAAGAAGGTGGTGAAGCAGG-3′ and reverse primer: 5′-TCAAAGGTGGAGGAGTGGGTGTCGC-3′), was used as a control for the amount of RNA. Real-time PCR assay was used to detect the expression levels of the Vimentin, N-cadherin and E-cadherin genes and the amplification was performed in triplicate. For each reaction, we prepared 1×SYBR Green Realtime PCR Master Mix (TOYOBO, Osaka, Japan), 1 μL forward primer and reverse primer and 1 μg of template cDNA in a final volume of 20 μL. The condition of the amplification were 45 cycles of sequential denaturation (95 °C, 2 min), annealing (60 °C, 15 s), and extension (72 °C, 20 s). The ΔΔCT value was used to analyze the rt-qPCR data. All samples were measured in triplicate experiments independently.

### 4.9. Statistical Analysis

Data are presented as Mean ± Standard deviation from triplicate experiments. All data were analyzed using One-way Analysis of Variance (ANOVA) for multiple comparisons by SPSS 16.0 (SPSS Inc., Chicago, IL, USA). Significant differences are indicated as follows: * *p* < 0.05; ** *p* < 0.01; *** *p* < 0.001.

## Figures and Tables

**Table 1:** Growth inhibitory activities of three actinomycins and Adriamycin in different cell lines.

Compounds IC_50_ (nmol/L)	MCF-7	MDA-MB-231	BT474	MCF-10A	HMLE
Actinomycin V	1.92 ± 0.25	0.83 ± 0.32	4.16 ± 0.25	4.07 ± 0.26	3.49 ± 0.31
Actinomycin D	8.23 ± 0.50	15.15 ± 0.52	37.00 ± 3.15	34.01 ± 0.25	30.22 ± 0.50
Actinomycin X_ob_	149.40 ± 4.03	127.33 ± 4.49	369.90 ± 0.14	248.57 ± 14.69	105.83 ± 8.44
Adriamycin	885.38 ± 13.50	942.60 ± 22.50	584.70 ± 50.00	1489.13 ± 25.50	1627 ± 15.50

^1^ The effects of three actinomycins and Adriamycin on various human breast cancer cells (MCF-7, MDA-MB-231, and BT474) and normal human breast epithelial cell lines (HMLE and MCF-10A) were examined by 3-(4,5-Dimethylthiazol)-2,5-diphenyltetrazolium bromide (MTT) assay. The cells were treated with various concentrations of compounds for 48 h and the IC_50_ values were then calculated. Results were obtained from three independent experiments. Data were expressed as the mean ± SD (Standard Deviation) of triplicate experiments.
